# Unexpected Long-Term Survival and Downstaging in Oligometastatic Non-Small Cell Lung Cancer Treated with Multimodal Therapy

**DOI:** 10.3390/jcm14103394

**Published:** 2025-05-13

**Authors:** Gabriela Rahnea-Nita, Nadejda Corobcean, Georgiana Bianca Constantin, Alexandru Nechifor, Adrian-Cornel Maier, Roxana-Andreea Rahnea-Nita, Dorel Firescu, Laura-Florentina Rebegea

**Affiliations:** 1 Specific Disciplines Department, Faculty of Nursery and Midwifery, Carol Davila University of Medicine and Pharmacy, 020021 Bucharest, Romania; gabriela.rahnea-nita@umfcd.ro; 2 M Hospital, 077190 Voluntari, Romania; roxana.rahnea-nita@umfcd.ro; 3 Oncology Department, “Nicolae Testemitanu” State University of Medicine and Pharmacy, 2004 Chisinau, Moldova; 4 Morphological and Functional Sciences Department, Faculty of Medicine and Pharmacy, Dunarea de Jos University, 800008 Galati, Romania; 5 Clinical Department, Faculty of Medicine and Pharmacy, Dunarea de Jos University, 800008 Galati, Romania; alexandru.nechifor@ugal.ro (A.N.); laura.rebegea@ugal.ro (L.-F.R.); 6 Surgical Department, Faculty of Medicine and Pharmacy, Dunarea de Jos University, 800008 Galati, Romania; adrian.maier@ugal.ro (A.-C.M.); dorel.firescu@ugal.ro (D.F.); 7 Clinical Department, Faculty of Medicine, Carol Davila University of Medicine and Pharmacy, 050474 Bucharest, Romania; 8 Radiotherapy Department, Clinical Emergency Hospital Sf. Ap. Andrei, 800216 Galati, Romania

**Keywords:** long-term survival, metastatic non-small cell lung cancer, multiple therapeutic lines, chemo-immunotherapy, radiotherapy

## Abstract

**Background**: Advances in the treatment of non-small cell lung cancer in the last 5 years (new techniques in radiotherapy, including stereotactic ablative radiotherapy, new targeted therapies and advances in immunotherapy) have increased the survival rates of patients diagnosed with this disease. **Methods**: Our study refers to a patient diagnosed in July 2017 with stage IV A lung cancer, cT3 N3 M1b (poorly differentiated adenocarcinoma, EGFR, ALK and PDL 1 negative), who underwent five lines of treatment and who has, at the time of writing this article (March 2025), a very good performance status, currently undergoing maintenance chemotherapy. **Results**: The results obtained confirm the revolutionary role of immunotherapy, but also the importance of chemotherapy and external radiotherapy, suggesting the synergistic effect between these three therapies. We also performed a literature review, highlighting the resistance to immunotherapy, rechallenge with immunotherapy, progression of metastatic NSCLC after first-line chemo-immunotherapy and the role of chemotherapy in line II and III after progression of NSCLC to immunotherapy. **Conclusions**: Results of studies evaluating new agents and their combinations, along with analysis of the mechanisms of evolution of primary and acquired resistance to immunotherapy are awaited, with the aim of selective and personalized treatment options to improve the survival and the quality of life for this category of patients.

## 1. Introduction

Lung cancer is the most common type of cancer and the leading cause of death in men and also the third most common after breast and colorectal cancer, and the second leading cause of death in women, after breast cancer [1,2].

Non-small cell lung cancer (NSCLC) represents 80–90% of all lung cancers [3].

The 5-year survival rate is 22.9% (localized disease 62.8%, regionally advanced 34.8%, metastatic only 8.2%), particularly 29.8% in NSCLC, compared to 5% 10–15 years ago [4].

Advances in treatment over the past 5 years (minimally invasive diagnostic and treatment techniques: new radiotherapy techniques, including stereotactic ablative radiotherapy (SABR); new targeted therapies and advances in immunotherapy) and screening have increased the 2-year survival rates to 42% in 2015–2016 from 34% in 2009–2010 [2,5,6,7,8].

Patients with NSCLC eligible for targeted therapies or immunotherapy usually survive longer now and the 5-year survival rates range from 15% to 50%, depending on the biomarker [4].

Combined chemotherapy (platinum doublet with third-generation molecules Gemcitabine/Vinorelbine or taxanes) is the first-line therapy in patients with metastatic NSCLC, without driver mutations, regardless of PD-L1 expression, having contraindications to immunotherapy and offers a 1-year survival of 40%. The prognosis of metastatic disease remains unfavorable, in conditions where targeted therapy or immunotherapy is not opted for.

Immunotherapy (IT) has revolutionized the treatment of metastatic lung cancer without driver mutations. Thus, monoimmunotherapy with ICI (anti-PD-1 Nivolumab or Pembrolizumab and anti-PD-L1 Atezolizumab) is the second-line therapy of choice in metastatic NSCLC, without driver mutations, regardless of PD-L1 expression, which progresses after the first-line therapy with chemotherapy.

In this context, the CheckMate 017 and 057 studies demonstrate a median survival of 11.1 months, a median duration of response of 19.9 months, and a 5-year survival rate of 82% in patients without progression after 2 years of treatment with Nivolumab [9].

Other recent studies demonstrate the following:

- After 12 months of treatment with Nivolumab, PFS increases to 38.1 months.
- Interrupting IT at 6 months in patients without progression or administering IT for 2 years maintains a prolonged progression-free survival (PFS) and long-term survival.

Radiotherapy, in addition to the local effect, can cause an abscopal effect, the mechanism of which is not very clear. Studies have shown that there is a connection between the effect of radiotherapy and the effect of immunotherapy. The combination of radiotherapy and immunotherapy acts at several stages of the tumor response, suggesting a synergistic effect of the two therapeutic modalities. This combination may also boost abscopal response or cure various cancers. It is about the activation of the immune system induced by radiotherapy, causing a specific type of apoptosis, the immunogenic cell death [10,11,12,13,14].

A study conducted in the laboratory revealed that the combination of radiotherapy and immunotherapy not only improves the immune microenvironment, but also has a positive effect on the prognosis of the disease [15].

The complexity of cancer requires planning and analysis to provide patients with a good quality of care, considering that both the disease and the side effects of treatments can negatively influence the quality of life [16,17,18].

The case we present is exceptional in terms of the number of therapeutic lines performed (five lines), downstaging from oligometastatic to advanced cancer and long-term survival (more than 7-and-a-half years).

## 2. Case Presentation

Our study refers to a 73-year-old patient, whose disease onset was in 07/2017 with intermittent dyspnea and pain in the right scapula region, with an ECOG performance status = 2.

Regarding the personal pathological history, the patient suffers from left ventricular congestive heart failure NYHA II, high blood pressure very high risk group, dyslipidemia, bicoronary atherosclerotic disease with a history of inferolateral myocardial infarction and infero-postero-lateral STEMI (05/2015) KILLIP with primary angioplasty with two BMS PE CD II stents, stent on ACD, mitral regurgitation grade II, bilateral pulmonary thromboembolism in 2017 and 2021, chronic kidney disease stage III, left renal parapyelic cyst, hepatic hemangioma, hypothyroidism (01/2019), benign prostatic hypertrophy, COVID infection in 2021 and right scapulohumeral periarthritis.

In his family history, the patient has an aunt with oncological disease.

Regarding his lifestyle, the patient is an amateur athlete, a former moderate smoker (10 cigarettes/day, for 30 years); former worker in a toxic environment for 20 years at the Galati Steel Plant (steel foundry) and 2 years of lead-welding activity.

**Figure 1:**
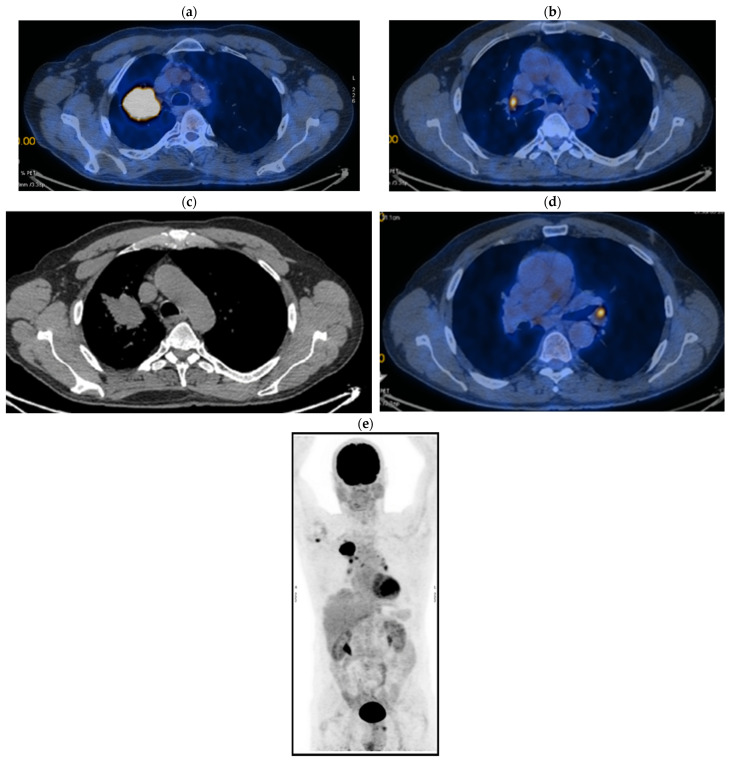
PET CT. (**a**) T—spiculiform LSD tumor formation of 44 × 57 × 63 mm with SUV = 12.59, with central necrosis; (**b**) the tumor invades the superior apical and mediastinal pleura; (**c**) N—metabolically active mediastinal ADP with SUV max = 7.43 located in the Ao-pulmonary window (14 × 9 mm), (**d**) infracarinal (12.8 mm) and bilateral hilar (right maximum 21 × 16 mm and left maximum 11 × 7 mm); (**e**) M—metabolically active focus with SUV = 5.6 right humeral head, which corresponds to a small interruption of the bone cortex.

His current treatment was Eliquis 2.5 mg, one tablet two times a day; Betaloc 50 mg, ½ tablet in the morning; Prestarium 5 mg, ½ tablet in the evening; Rosuvastatin 10 mg, one tablet in the morning; Aspenter 75 mg, one tablet/day; Euthyrox 50 mg, one tablet/day; Urorec 8 mg and one tablet/day. The staging imaging (PET-CT) from 2017 revealed the following images (as shown in Figure 1a–e):

In July 2017, a thoracotomy with biopsy (right upper lobe and visceral pleura) was performed. Histopathological and immunohistochemical examinations revealed poorly differentiated adenocarcinoma and PDL 1, ALK and EGFR tests were negative.

The definitive diagnosis is right bronchopulmonary tumor (right superior lobe), extended to the visceral pleura and bilateral mediastinal lymph nodes. A palliative surgery 07/2017–thoracotomy with biopsy (from LSD and visceral pleura) was performed. The histopathological examination revealed a poorly differentiated adenocarcinoma, ALK, EGFR, PDL 1 negative, cT3 N3 M1b (OSS, single, right humeral head) and stage IVA.

The patient started the systemic first-line therapy–palliative chemotherapy 3xCis/Gem (08–12/2017) + switch 3xCarbo/Gem (07/02–04/2018).

The patient was biologically evaluated and the imaging evaluation was quantified according to RECIST criteria.

Reevaluation of CT 05/12/2017 versus PET CT of 05/07/2017 revealed a partial response, according to RECIST criteria (primary tumor reduction by 45.5%: 39 × 16 vs. 44 × 57 mm and mediastinal and hilar lymph nodes by 52.3%: 10 vs. 21 mm). The Oncology Committee (12/2017) recommended a switch to (3xCarbo/Gem, 07/02–23/04/2018) due to grade III neutropenia + bisphosphonates (Zoledronic Acid).

**Figure 2:**
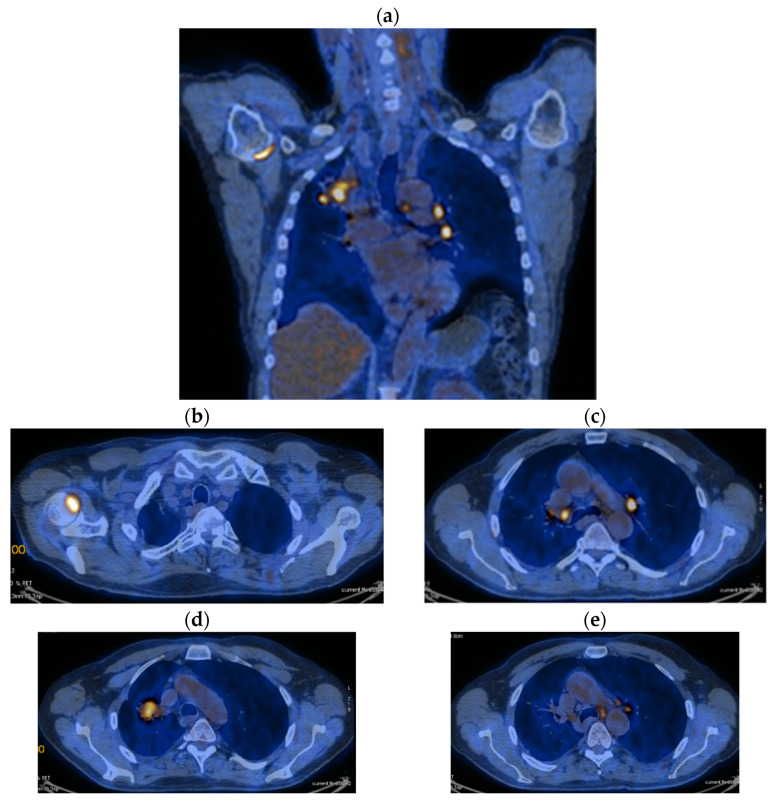
(**a**–**e**) PET CT reevaluation.

Reevaluation of PET CT 06/03/2018 vs. PET CT from 07/2017 highlighted stable disease (primary tumor regression by 12.87%: 29 × 59 mm vs. 44/57/63 and metabolic: SUV = 7.89 vs. 12.59) and metabolic progression on bilateral mediastinal and hilar lymph nodes: SUV 8.8 vs. 7.43) and M1OSS (right humeral head): SUV 6.04 vs. 5.6) (Figure 2a–e).

Considering the osseous pain and functional impotence with limited movements in the scapula–humeral joint, we decided to perform palliative radiotherapy on the right humeral head PTV: DT = 30 Gy/10 on 04–05/2018, with improvement of the painful symptoms.

The patient began second-line systemic therapy (Nivolumab) on 05/25/2018 and underwent this treatment until 04/2020 (1 year 11 months = 23 months), with stable disease at the level of the primary tumor and mediastinal lymph nodes until June 2019, when disease progression was recorded. The case was discussed in the Oncology Committee and it was decided to stop Nivolumab. Reevaluations were performed every 3 months, from May 2018 to April 2020.

Reevaluation of CT from 08/29/2018 vs. CT from 12/2017 highlighted disease progression (primary tumor growth by 21.81%: 43 × 24 mm vs. 39 × 16) and vs. PET CT from 03/2018: disease progression (primary tumor growth by 23.86%: 43 × 24 vs. 29 × 59 mm) and stable disease on lymph nodes.

**Figure 3:**
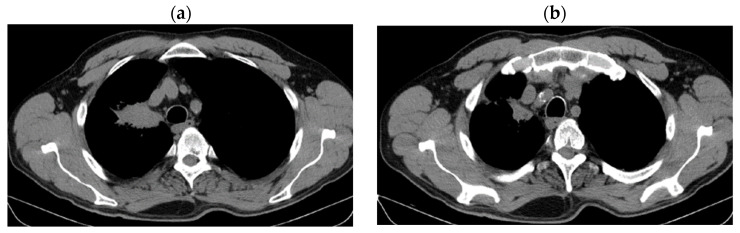
(**a**,**b**) Stable disease after 6 months of Nivolumab.

Reevaluation of CT from 11/29/2018 vs. CT from 12/2017 highlighted stable disease (after 6 months of Nivolumab) on the primary tumor (43 × 22 vs. 43 × 24 mm) and progression on lymph nodes (15 mm vs. 6 mm), without detection of M1OSS (as seen in Figure 3a,b).

Reevaluation of CT from 01/03/2019 vs. CT from 11/2018 highlighted stable disease (primary tumor growth by 15.38%: 24 × 51 vs. 43 × 22) and lymph nodes (16 vs. 15 mm), without M1OSS. Oncological Committee no. 217/12.03.2019 decided to continue Immunotherapy (until 13/03/2020).

The CT reevaluation from 04/06/2019 vs. CT from 03/2019 revealed stable disease (primary tumor growth by 5.34%: 29 × 49 × 60 vs. 24 × 51 × 56 mm) and mediastinal and hilar lymph nodes (16 mm), without M1 OSS and a pruritic acneiform rash.

**Figure 4:**
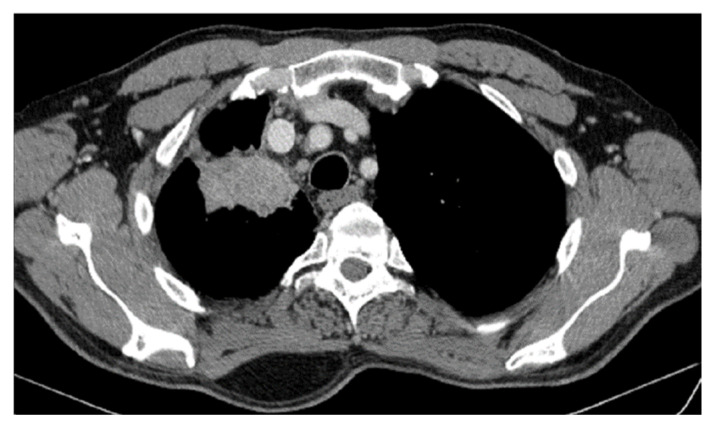
Progression after 23 months of Nivolumab.

CT reevaluation from 04/2020 vs. CT from 06/2019 revealed disease progression (primary tumor growth by 34.78%: 45 × 64 × 77 vs. 29 × 49 × 60) and stable disease on mediastinal and hilar lymph nodes (Figure 4).

**Figure 5:**
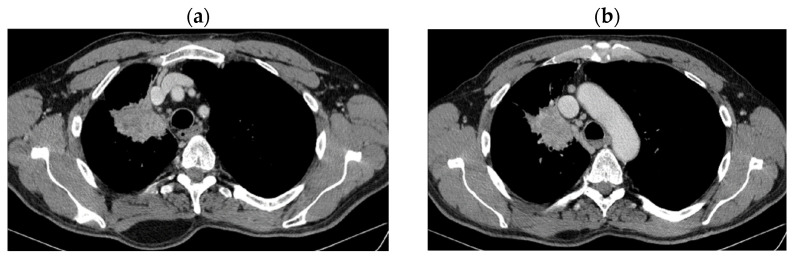
Progression of the disease: (**a**) Thoracic CT hilar nodes; (**b**) mediastinal nodes.

The Oncological Committee 551/04/2020 decided to stop Nivolumab, considering the evolution of the disease (Figure 5a,b).

### 2.1. Systemic Third-Line Therapy (4xDocetaxel) Was Initiated 04–07/2020

Definitive radiotherapy was integrated on PTV—primary tumor + regional lymph node areas (hilar, parahilar, SCV) with a total dose (TD) = 50.4 Gy/28 fractions/5 weeks with a boost on the primary tumor up to DT = 61.2 Gy − 05.08–18.09.2020. The patient had good compliance with the treatment, without hematological toxicities with grade I esophageal toxicity on the CTCEAVS5 scale.

**Figure 6:**
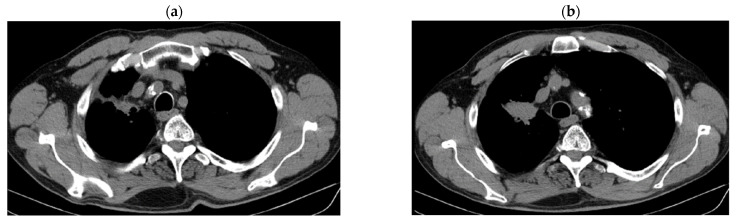
(**a**,**b**) Reevaluation CT from 02/11/2020 vs. CT from 04/2020.

CT reevaluation from 02/11/2020 vs. CT from 04/2020 revealed stable disease at the borderline with partial response (primary tumor reduction by 29.03%: 32 × 45 × 55 vs. 45 × 64 × 77 mm) and stable disease on mediastinal and hilar lymph nodes, without M1 OSS (Figure 6a,b).

CT reevaluation from 05/14/2021 vs. CT from 11/2020 revealed stable disease (primary tumor regression by 12.87%: 40 × 25 × 50 vs. 32 × 45 × 55 mm) and stable disease on mediastinal lymph nodes (14 vs. 16 mm).

**Figure 7:**
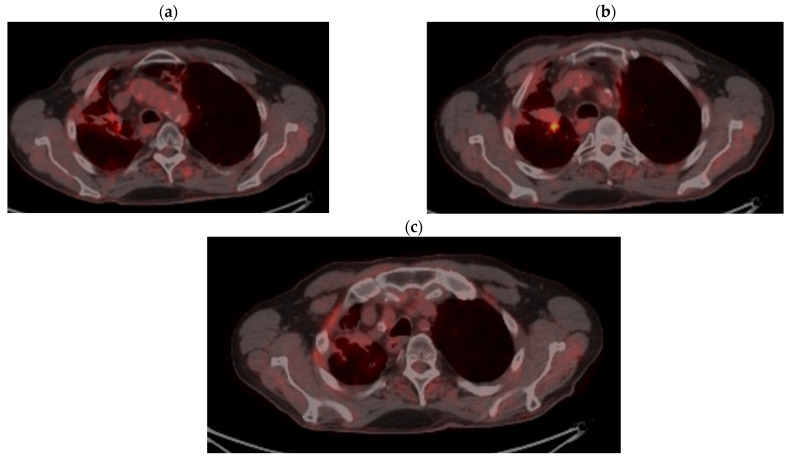
(**a**–**c**) Reevaluation of PET CT from 11/17/2021 vs. PET CT from 03/2018.

PET CT reevaluation from 11/17/2021 vs. PET CT from 03/2018 showed stable disease (primary tumor regression by 15.8%: 28 × 34.2 vs. 29 × 59 mm with metabolic regression: SUV = 5.76 vs. 7.89 and regression of mediastinal and hilar lymph nodes (15.1 vs. 16 mm and metabolic SUV = 7.45 vs. 8.08. Left hilar lymph node 12 × 10.6 mm with SUV = 10.88) and metabolic regression of M1 OSS: SUV = 4.28 vs. 6.04 (Figure 7a–c).

Systemic Fourth-Line Therapy (5xCARBO/PACLI) was initiated (16/01–20/04/2021).

### 2.2. Monitoring 2022: Oncological Commission 1910/16.08.2022

CT reevaluation from 11/02/2022 = CT from 05/2021 highlighted stable disease. Reevaluation of CT from 10/06/2022 vs. CT from 02/2022 highlighted stable disease (primary tumor increase by 9.56%: 26 × 56 × 44 vs. 40 × 25 × 50 mm) and on mediastinal lymph nodes.

**Figure 8:**
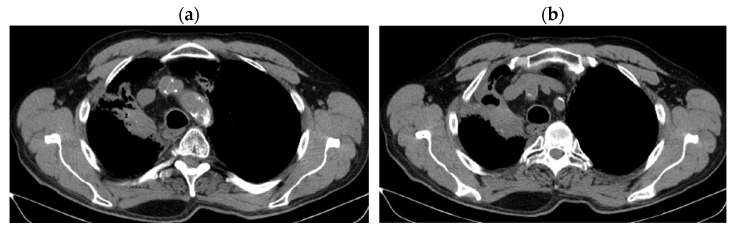
(**a**,**b**) CT reevaluation from 23/09/2022 vs. 06/2022.

The CT reevaluation from 23/09/2022 vs. 06/2022 highlighted, again, stable disease (primary tumor reduction by 12.69%: 24 × 46 × 40 vs. 26 × 56 × 44) and on mediastinal lymph nodes) (as showed in Figure 8a,b).

The disease was stable for 2 years and 4 months = 28 months (11/2020–03/2023), the patient being left without treatment since 04/2021 (1 year and 7 months = 19 months).

**Figure 9:**
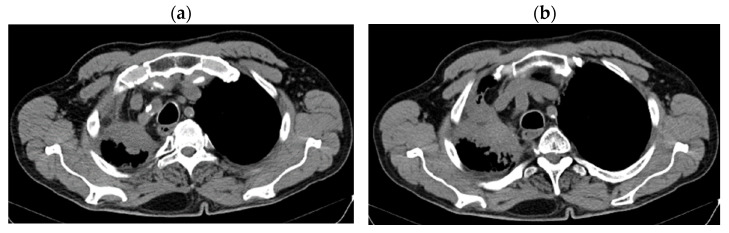
(**a**,**b**) CT from 30/03/23 vs. CT from 09/2022: DISEASE PROGRESSION (primary tumor growth by 22.32%: 33 × 51 × 53 vs. 24 × 46 × 40 mm).

**Figure 10:**
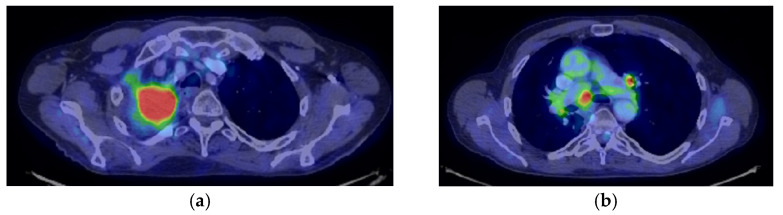

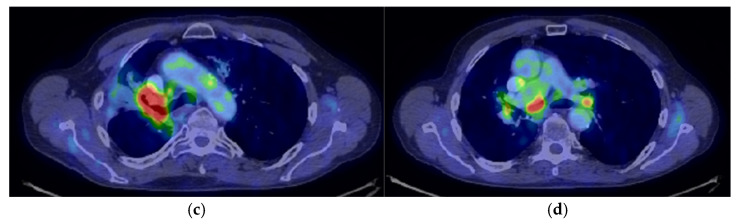
(**a**–**d**) PET CT from 06/14/2023 vs. PET CT from 11/2021: disease progression (primary tumor growth by 31.83%: 42 × 40 vs. 28 × 34.2 mm) and metabolic progression at SUV = 15.13 vs. 5.7; dimensional and metabolic regression on bilateral hilar lymph nodes (14 vs. 15.1 mm; SUV = 5.52 vs. 10.88), dimensional and metabolic progression of infracarinal lymph node (29 vs. 12 mm; SUV = 8.48 vs. 5.95 at PET CT from 03/2018, because they are not described at PET CT from 11/2021), without pathological uptake at the level of the scanned bone segments.

Disease progression was recorded from 03/2023, 35 months (2 years, 11 months) after the interruption of immunotherapy (04/2020–03/2023) and 23 months after the completion of the fourth-line therapy (04/2021–03/2023) (as seen in Figure 9a,b and Figure 10a–d).

### 2.3. Oncological Committee 07/25/2023 Recommended

Prebiopsy of the primary tumor or/and mediastinal lymph nodes for histopathological and immunohistochemical examination, with subsequent testing of PDL1, EGFR, ALK, ROS, BRAF for targeted therapy. A bronchoscopy with rebiopsy was practiced on 08/16/2023 and histopathological report and IHC tests from 08/2023 reconfirmed poorly differentiated adenocarcinoma, ALK-negative EGFR-negative and PD-L1 low expression (TPS = 5%).

The Oncological Committee of 11/2023, taking into account the disease progression confirmed by CT imaging of 03/2023 vs. 09/2022 and PET CT of 06/2023 vs. 11/2021 with primary tumor growth by over 20% and pathologically certain SUV, recommended the following. Re-initiation of systemic therapy: fifth-line therapy with Pemetrexed/Platinum salt + Navelbine + CT/PET CT imaging reassessment of tumor response at 3 and 6 months, which the patient delayed having an IP 0 (ECOG). Then, the systemic fifth-line therapy (Pemetrexed maintenance 11.2023–02.2025) was administered.

According to the recommendations of the Oncological Committee from November 2023, chemotherapy with Pemetrexed 500 mg/m^2^ was continued, the first cycle being administered in November 2023 and the patient continued this treatment throughout 2024, until February 2025, the date of writing this article.

The definitive diagnosis was right bronchopulmonary tumor (upper segments LSD, extended to visceral pleura). Palliative surgery 07/2017—Thoracotomy with biopsy (LSD and visceral pleura). Mediastinal lymph nodes (Ao-pulmonary window, infracarinal) and bilateral hilar. Secondary lesion right humeral head. HP: poorly differentiated adenocarcinoma, ALK, EGFR negative, cT3N3M1b (OSS, single, right humeral head), stage IVA.

To summarize: Systemic first-line therapy (6xCis/Carbo + Gem (08–12/2017) with stable disease and metabolic progression bilateral mediastinal and hilar lymph nodes and M1OSS. Palliative RTE (right humeral head) DT = 30 Gy (04–05/2018). Second-line therapy (05/2018–04/2020) with disease progression 04/2020 (23 months). Third-line therapy: 4xDoce (04–07/2020) with stable disease 11/2020. Palliative RTE (primary tumor + regional lymph node areas) DT = 61.2 Gy (05.08–18.09.2020). Fourth-line therapy: 5xCarbo/Pacli (01–04/2021) with stable disease 11/2021. Imaging follow-up 02/2022–09/2022 confirmed stable disease. Disease without progression 11/2020–03/2023 (2 years and 4 months = 28 months). Without oncological treatment, 1 year and 7 months (04/2021–11/2023). Disease progression 03/2023 (CT) and 06/2023 (PET CT). Rebiopsy (bronchoscopy) 16/08/2023 (HP + IHC + Genetic tests: poorly differentiated adenocarcinoma; ALK, EGFR, PD-L1 positive) low expression (TPS = 5%). Restaging: rT2bN3M0 (without detection of M1 OSS), stage IIIB. Fifth-line therapy: maintenance with Pemetrexed (11.2023–02.2025). Table 1 summarizes the data.

The patient’s performance status is currently ECOG = 1. Hematological toxicities during the treatment lines were grade 1 and 2. Febrile neutropenia prophylaxis was per-formed. During immunotherapy, the only immune-mediated toxicity was hypothyroidism, which occurred after 6 months, for which thyroid replacement treatment with Euthyrox was initiated.

Alternative methods:

- Consider rescue re-irradiation (SBRT), taking into account the current primary tumor dimensions of 24 × 34.2 mm on PET CT from 06/2023 and also the total dose previously administered (08–09/2020), the previously applied fractionation, the time between the two irradiations, the cellular repair time and the dose constraints for the organs at risk.
- Genetic testing for ROS1, BRAF V600E, NTRK1/2/3, MET Exon14, RET and ERBB2 (HER2) in order to consider the opportunity for targeted therapy.

## 3. Case Results

The stable disease, with no progression (between 11/2020 and 03/2023) was achieved after stopping NIVO. The patient had no oncological treatment for 19 months (04/2021–11/2023). The detailed evolution is presented in Table 2.

## 4. Case Conclusions

Although stopped due to disease progression after 23 months, Nivolumab immunotherapy, followed by two subsequent lines of chemotherapy (Docetaxel monotherapy and Carbo/Pacli combination), in association with radiotherapy on the primary tumor, regional lymph node areas and metastatic bone lesion, maintained the therapeutic response (stable disease) for 28 months (2 years and 4 months), with a survival from the onset of IT of 6 years and 9 months, with an overall survival of 7 years and 7 months, with downstaging from metastatic disease to locoregional disease, with the maintenance of a good performance status, subsequently with disease progression, for which maintenance chemotherapy (Pemetrexed) was resumed.

These results confirm the revolutionary role of IT, but also the importance of chemotherapy and external radiotherapy, suggesting the synergistic effect between these three therapies.

## 5. Discussion and Literature Review

The choice of subsequent therapy in NSCLC patients with oligometastatic disease and good performance status who have progressed after first-line treatment presents a challenge. We present below immunotherapy resistance, rechallenge with immunotherapy, progression of metastatic NSCLC after the first line of chemo-immunotherapy and the role of chemotherapy in second- and third-line therapies after progression of NSCLC to IT.

A multidisciplinary approach plays a crucial role in establishing the diagnosis and developing a personalized treatment plan in lung cancer patients.

Important questions remain regarding the optimal treatment regimen, identifying patients who may benefit from adding chemotherapy and/or anti-CTLA4 antibodies to anti-PD-(L)1 antibodies and predictive biomarkers [19].

### 5.1. Immunotherapy Resistance

Within 5 years, 74% of NSCLC patients with an initial effective response to immunotherapy will progress [20].

The mechanism of resistance (primary and acquired) to immunotherapy is complex, dynamic and interdependent, mainly involving intrinsic factors, tumor cells, and extrinsic factors, immune cells, infiltrated in the tumor microenvironment (TME) [21].

Radiotherapy, chemotherapy, anti-angiogenesis drugs and TME modulators can synergistically enhance immunotherapy by regulating the cancer-immunity cycle process, including tumor antigen release, presentation and immune cells infiltrated in the TME. These synergistic effects are uncontrollable and unpredictable [22].

Transformation of NSCLC into SCLC is a recognized mechanism of resistance to EGFR-TKI agents in the treatment of EGFR-mutated NSCLC, with a rate of 3–14%. Patients with SCLC may present with paraneoplastic syndromes, with a short overall survival without treatment (4–6 months) [23].

The best response to immunotherapy with Nivolumab was partial response or stable disease. Rebiopsy was performed by EBUS with transbronchial biopsy or CT-guided fine-needle biopsy of both the primary tumor and the progressive lymph nodes in all cases. The time-period from the start of immunotherapy to histological confirmation of small cell transformation varies between 2 weeks and 3 years [20,21,22,23,24,25].

The results of studies evaluating new agents and their combinations, along with the analysis of the mechanisms of evolution of primary and acquired resistance to immuno-therapy and the identification of potential biomarkers, are awaited for the purpose of selective and personalized treatment options to improve survival and quality of life for this category of patients [24,25].

Recent data suggest that the mode of progression after resistance to immunotherapy should be considered for continued treatment [24].

### 5.2. Rechallenge with Immunotherapy

Retreatment with PD-1 inhibitor (Nivolumab or Pembrolizumab) has been reported in clinical trials that included a limited number of patients [24,25].

Rechallenge with the same ICI in NSCLC patients who received treatment with Pembrolizumab for 2 years, showed a clinical benefit at disease relapse [26].

Retreatment with pembrolizumab in the KEYNOTE-010 study of platinum pretreated patients with PD-L1 ≥ 1% led to an objective response in 11 out of 21 patients (52.3%) per RECIST by independent central review (complete response, *n* = 1; partial response, *n* = 10) after starting second-course pembrolizumab. A further six patients had stable disease, for an overall disease control rate of 81.0%. In addition, three patients had progressive disease per RECIST by independent central review and one patient was unevaluable. Eight patients experienced subsequent disease progression per irRC (immune-related response criteria) by investigator assessment, including three patients who had achieved a partial response and five who had stable disease [24].

In the KEYNOTE-024 trial of chemo naïve patients with PD-L1 ≥ 50%, a partial response or stable disease was obtained in 10 out of 12 patients (83%). In many cases, the efficacy of immunotherapy is limited, and it is necessary to determine the optimal sequence of systemic treatment. There is some theoretical rationale for repeated use of immune checkpoint inhibitors, but it is debatable which subgroups of patients are likely to benefit clinically from such treatment. There is a need for research identifying factors that will guide clinical decision-making, such as the time from the completion of immunotherapy, the initial response achieved, the expression of PD-L1 and others [25,26].

Several retrospective studies have shown a survival of more than one year from the time of ICI rechallenge. This survival was greater in patients who discontinued ICI treatment because of toxicity compared with patients who discontinued ICI treatment because of disease progression. Thus, it appears that patients who discontinued immunotherapy due to disease progression should not be requalified for treatment with currently available ICIs [27]. 

Several studies have suggested that radiation therapy, especially stereotactic body radiotherapy (SBRT), may have a synergistic role in the treatment of metastatic lung cancer, after immunotherapy failure [28,29].

Rechallenging immunotherapy is feasible and patients should be carefully evaluated before initiating treatment and being monitored for benefits/risks [30,31].

Furthermore, to choose the most appropriate treatment and to understand the mechanisms leading to resistance to ICI treatment, rebiopsy at baseline is necessary [32,33].

These data suggest that rebiopsy and molecular re-evaluation may guide rechallenge strategies in selected patients.

### 5.3. Progression of Metastatic NSCLC After First Line of Chemo-Immunotherapy

Pivotal clinical trials report that 30% up to 46% of patients are able to receive further therapies after chemo-immunotherapy [32].

For patients with advanced NSCLC without molecular aberrations, whose tumor progresses after first-line chemo-immunotherapy, standard options of treatment include Docetaxel, Pemetrexed or Gemcitabine, or Docetaxel in combination with antiangiogenic agents such as Nintedanib or Ramucirumab [34,35,36].

Recent data suggest that prior treatment with immunotherapy could confer a synergistic benefit to subsequent chemotherapy and therefore to increase efficacy [37].

New combinations or therapeutic agents are currently being tested as a second-line therapy after chemo-immunotherapy to improve outcomes.

- Combination with angiogenesis inhibitors: Ramucirumab/Pembrolizumab+Docetaxel [38].
- Combination with Trophoblast cell surface antigen (TROP2): Sacituzumab govitecan+Docetaxel; Datopotamad deruxtecan; Tusamitamab ravtansin [39,40,41].
- Combination of anti-PD-1 + anti-T-cell immunoglobulin and ITIM domain: Docetaxel + anti-PD-1 alone or in combination + anti-T cell immunoglobulin and mucin-domain containing-3 [42,43].
- Combination between cytokines such as TGF-B, IL-1b, IL-15, or CXCR2 + either Docetaxel or ICI [44,45].

Data suggest that these strategies share the aim of enhancing the immune response.

### 5.4. The Role of Chemotherapy in Second-Line and Third-Line Therapies After Progression of NSCLC to IT

To date, very few studies have explored the efficacy of second- or third-line chemotherapy after progression following first-line chemo-ICI, and the studies highlight the need for new combinations and strategies in second-line after progression following first-line combination of chemo-ICI [36,37].

A multicentric international study retrospectively included patients with advanced NSCLC with progressive disease, who had undergone first-line chemo-ICI (anti-PD-1 alone or anti-PD-1 in combination with anti-CTLA-4) regimens and were eligible for second-line treatments. In total, 57 (46.0%) patients received taxane monotherapy, 25 (20.1%) taxane plus anti-angiogenic, 12 (9.7%) platinum-based chemotherapy and 30 (24.2%) other chemotherapy. The median first-line treatment overall survival (OS) was 8.1 months and the median second-line treatment PFS was 2.9 months. The results highlight a significant difference of first-line treatment responses with a median OS of 5.1 months for first-line resistant patients and 12.7 months for the responders. Taxane plus anti-angiogenic and platinum rechallenge achieved the longest median second-line OS: not reached and 17.6 months, respectively. Patients resistant to the treatment had inferior outcomes (first-line OS 5.1 months, second-line PFS 2.3 months) compared with first-line responders (second-line OS 12.7 months, second-line PFS 3.2 months) [39].

Thus, second-line chemotherapy after progression following first-line chemo-ICI achieved modest activity in patients with NSCLC. Rechallenge with platinum-based chemotherapy or taxane plus anti-angiogenic may be an effective option for selected populations. Patients with tumors resistant to first-line treatments remain a refractory population that requires to be enrolled in clinical trials [40,42]. 

The clinical improvements provided by first- and second-line treatment in NSCLC have led a higher proportion of patients to be considered for third-line treatment [43,44]. 

In a retrospective study made in Lebanon, based on a chart review of patients diagnosed with advanced NSCLC who received Docetaxel as second- or third-line after being treated by immunotherapy and/or chemotherapy in previous lines, the authors reported a 24% response rate to Docetaxel including stable disease and partial response and a median progression free survival (PFS) of 3 months [45].

Despite the discovery of new immunotherapy and targeted therapy drugs, recent data suggest that chemotherapy is still an option for the treatment of advanced NSCLC after progression on immunotherapy alone or in combination with first-line chemotherapy [45].

## 6. Conclusions

Immunotherapy in advanced NSCLC without driver mutations has demonstrated remarkable benefits in overall survival and progression-free survival, with unprecedented long-term objective response rates. However, most patients do not benefit from IT or relapse after a period of response, due to the development of primary and secondary resistance.

One of the mechanisms of resistance to immunotherapy is the transformation of NSCLC into SCLC, the frequency of which is underestimated, given the lack of widespread practice of rebiopsy in case of progression, which must always be taken into account.

Yet, there are no data available on the potential role of resuming immunotherapy in patients with NSCLC without driver mutations who have progressed after chemo-immunotherapy. Taking into account the heterogeneity of patients depending on the first-line therapy administered, and the reasons for previous interruption of immunotherapy and chemotherapy in the second and third lines, retains its important role in disease control, survival and quality of life.

Thus, the patients with advanced NSCLC without driver mutations, who progress after chemo-immunotherapy, are a current clinical challenge, considering the modest results offered by the standard options available. In this context, the decision for the second-line therapy must consider the inclusion of patients in clinical trials, to provide the best treatment and to increase the availability of data on new therapeutic options.

## Figures and Tables

**Table 1:** Patient’s results after the five lines of therapy.

Period of Time	Treatment	Regimen
08–12/2017	First-line therapy	6xCis/Carbo + Gem
04–05/2018	Palliative RTE (right humeral head)	DT = 30 Gy
05/2018–04/2020	Second-line therapy	
04–07/2020	Third-line therapy	4xDoce
05.08–18.09.2020	Palliative RTE (primary tumor + regional lymph node areas)	DT = 61.2 Gy
01–04/2021	Fourth-line therapy	5xCarbo/Pacli
No oncological treatment. Stable disease		
11.2023–02.2025	Fifth-line therapy	Pemetrexed

**Table 2:** Patient’s evolution during the entire oncological treatment.

Results	Details	Time	Period
Progression of disease on IT	Secondary resistance at NIVO	At 23 months (1 year and 11 months) of NIVO	05/2018–04/2020
Survival without progression	Stable disease	28 months (2 years and 4 months)	11/2020–03/2023
Progression after CHT + IT	From Stop NIVO	At 35 months (2 years and 11 months)	04/2020–03/2023
	From finishing Line IV	At 23 months (1 year and 11 months)	04/2021–03/2023
Without oncological treatment		1 year and 7 months	04/2021–11/2023
Survival	Since starting NIVO	6 years and 9 months	05/2018–02/2025
	Since stopping NIVO	4 years and 10 months	04/2020–02/2025
	After Line IV (CHT)	4 years and 1 month	04/2021–02//2025
	After Line V (CHT)	1 year and 3 months	11/202302/2025
General survival		7 years and 7 months	07/2017–02/2025
IP 0 (ECOG)	Continues professional activity	02/2025	
DOWNSTAGING from metastatic to locoregional stage	From cT3N3M1b(OSS) stage IVA	In cT2bN3M0, stage IIIB (without M1OSS)	

## Data Availability

Data are contained within the article.
